# Lack of adipocyte FAM20C improves whole body glucose homeostasis

**DOI:** 10.14814/phy2.70126

**Published:** 2024-11-12

**Authors:** Liping Deng, Yanshan Huang, Feifei Zhao, Puxin Chen, Xiaohong Huang

**Affiliations:** ^1^ Department of Endocrinology Longgang District Central Hospital of Shenzhen Shenzhen Guangdong China; ^2^ Division of Preventive Health Longgang District Central Hospital of Shenzhen Shenzhen Guangdong China; ^3^ Department of Nursing School of Medicine, Shantou University Shantou Guangdong China; ^4^ Department of Nursing Shenzhen Clinical Medical College, Guangzhou University of Chinese Medicine Shenzhen Guangdong China; ^5^ Department of Nursing Longgang District Central Hospital of Shenzhen Shenzhen Guangdong China

**Keywords:** adipose tissue, FAM20c, inflammation, insulin resistance, obesity

## Abstract

FAM20C, a member of the family with sequence similarity 20, is involved in many physiological functions. Obesity, characterized by excessive accumulation of adipose tissue, has attracted more and more attention as a worldwide health problem. Here we generated adipocyte‐specific FAM20C knockout mice to investigate the role of FAM20C in adipose tissue expansion and obesity. Our results demonstrate that knockout mice are protected against high fat diet‐induced obesity, adiposity, and fatty liver disease. Additionally, knockout mice exhibited improved metabolic phenotypes, including enhanced glucose tolerance and insulin sensitivity compared with control mice. Furthermore, we observed reduced inflammatory infiltration and collagen deposition in the adipose tissues of knockout mice. Taken together, our results indicate that targeting FAM20C in adipocytes may be a promising strategy for the treatment of obesity and associated metabolic disorders.

## 1 INTRODUCTION

Obesity is a significant global healthcare challenge and a primary risk factor for cardiometabolic diseases, including diabetes, nonalcoholic steatohepatitis, and cardiovascular disease (Gonzalez‐Muniesa et al., 2017; Neeland et al., 2019). The expansion of adipose tissue in obesity occurs through two primary processes: adipocyte hypertrophy, characterized by an increase in the size of adipocytes, and adipocyte hyperplasia, which involves the recruitment and development of preadipocytes, leading to a higher number of adipocytes (Spalding et al., 2008). The relationship between adipose cell size and obesity is non‐linear. When the number of adipocytes reaches a plateau, new adipocyte generation is triggered. While adipocyte hyperplasia acts as a protective mechanism against metabolic dysfunction, adipocyte hypertrophy is associated with adipose tissue dysfunction and the development of insulin resistance (Krotkiewski et al., 1983; Ryden et al., 2014). Hypertrophic adipocytes contribute to insulin resistance through mechanisms such as endoplasmic reticulum (ER) stress and pathways involving inflammation, oxidative stress, and interactions with immune cells (Hammarstedt et al., 2018). Although obesity increases the risk of comorbidities, some individuals with obesity do not develop related health issues (Appleton et al., 2013; Denis & Obin, 2013; Voulgari et al., 2011), highlighting the complex nature of adipose tissue and its metabolic interactions. Evidence suggests that anti‐apoptotic mechanisms support adipocyte survival in expanding adipose tissue (Monji et al., 2022). However, the precise mechanisms regulating adipocyte survival during adipose tissue expansion remain unclear.

The FAM20 family is comprised of three main members: FAM20A, FAM20B, and FAM20C. FAM20A is predominantly expressed in the liver and lung (Nalbant et al., 2005), while FAM20B has been implicated in skeletal development (Eames et al., 2011). FAM20C, the most extensively researched member, is a secretory protein with kinase activity that is ubiquitously expressed across various tissues, including mineralized zones (Tagliabracci et al., 2015; Wang et al., 2012; Xu et al., 2021). FAM20C plays an essential role in regulating cell motility, proliferation, and survival. Previous studies have focused on the function of FAM20C in the regulation of skeletal‐related organ development (Liu et al., 2017, 2018) and tumorigenesis (Zuo et al., 2021). However, the role of FAM20C in metabolic diseases and tissue growth, such as in adipose tissue, is understudied in comparison with other tissues and cell types.

In this study, we generated mice with adipocyte‐specific deletion of FAM20C. We discovered that mutant mice on a high‐fat diet, but not a normal chow diet, exhibited reduced adiposity, enhanced insulin sensitivity, and decreased hepatic steatosis during obesity. The deficiency of FAM20C in adipocytes resulted in reduced adipose tissue expansion, primarily by alleviating adipocyte inflammation.

## 2 MATERIALS AND METHODS

### 2.1 Animal studies

The *Fam20c* ^*flox/flox*^ mouse model was previously characterized (Wang et al., 2012). *Adiponectin‐Cre* transgenic mice were purchased from the Jackson Laboratory (Lee et al., 2013). To generate mice with adipocyte‐specific deletion of Fam20c, *Fam20c* ^*flox/flox*^ mice were crossed to transgenic mice expressing Cre recombinase driven by the adiponectin gene promoter to get *Adiponectin‐Cre; Fam20c* ^*flox/+*^ mice. Male *Adiponectin‐Cre; Fam20c* ^*flox/+*^ mice were then crossed to female *Fam20c* ^*flox/flox*^ mice to obtain homozygous *Adiponectin‐Cre; Fam20c* ^*flox/flox*^ mice (referred to as KO mice). *Fam20c* ^*flox/flox*^ mice were used as littermate control (referred to as flox). All mice were backcrossed with C57BL/6 mice for more than 10 generations. Only male mice were used for experiments and the littermate controls were used. For HFD experiments, mice were fed with high fat diet containing 60% kcal fat (Research Diets; D12492) for 12 weeks and body weights were monitored weekly. Male ob/ob mice aged 8–12 weeks were purchased from GemPharmatech Company. All mice were housed in groups under controlled temperature conditions (20–24°C) and subjected to a 12‐h light/12‐h dark cycle. The animal experimental protocol was approved by the Animal Care Committee of Shenzhen University.

### 2.2 Insulin tolerance test and glucose tolerance test

Insulin tolerance test (ITT) and glucose tolerance test (GTT) were performed as previously described (Xiao et al., 2013). For the GTT, mice were fasted overnight and then administered an intraperitoneal injection of glucose at a dosage of 1.5 g/kg. Blood glucose levels were measured at indicated time points post‐administration. For the ITT, mice were fasted for 4 h before receiving an intraperitoneal injection of insulin at a dose of 1 U/kg body weight, with blood glucose levels measured at the indicated time points.

### 2.3 Serum biochemical analyses

Serum insulin levels were measured using ELISA kits from Millipore. Non‐esterified fatty acid (NEFA) and triglyceride (TG) concentrations were measured using kits according to the manufacturer's instructions.

### 2.4 SDS–PAGE and western blotting

Tissues were homogenized in RIPA lysis buffer supplemented with protease and phosphatase inhibitors. Protein samples (30 μg) were separated by SDS‐PAGE and transferred onto PVDF membranes. The membranes were blocked with 5% non‐fat milk in Tris‐buffered saline/Tween 20 buffer at room temperature for 1 h, followed by overnight incubation at 4°C with primary antibodies. Membranes were then incubated with secondary antibodies and visualized using an ECL commercial kit. Primary antibodies used were: anti‐FAM20C (ab154740, Abcam, Cambridge, UK) and anti‐tubulin (TA506581, ZSGB‐BIO, China).

### 2.5 Histological analysis

Mouse white adipose tissue and liver samples were fixed in 4% paraformaldehyde (PFA) for 24 h at room temperature. Tissues were then embedded in paraffin, sectioned at 5 μm, and subjected to hematoxylin and eosin (HE), immunohistochemical, and immunofluorescence staining. Sections were deparaffinized and rehydrated before H&E staining. Nuclei were stained with hematoxylin for 5 min and eosin for 2 min. Digital images were acquired using a microscope scanner. For immunohistochemistry and immunofluorescence staining, sections were deparaffinized, antigen‐retrieved, blocked for 1 h, and incubated overnight with primary antibodies (Table S1). After PBS washing, sections were incubated with secondary antibodies for 45 min at room temperature.

### 2.6 Indirect calorimetry

The metabolic cage test was performed 1 week prior to sacrificing the mice. Food and water intake, carbon dioxide production (VCO_2_), oxygen consumption (VO_2_), and energy expenditure were measured by using a comprehensive laboratory animal metabolic system (Columbus Instruments).

### 2.7 Oil red O staining and liver TG quantification

Frozen liver sections were prepared and stained with Oil Red O stock solution diluted with ddH2O (3:2, v/v) for 30 min. Liver samples were homogenized, lipid‐extracted using a chloroform: methanol (2:1) solution, dried by evaporation, and quantified using a commercial kit as per the manufacturer's instructions.

### 2.8 RNA extraction and real‐time PCR analysis

Total RNA was extracted using Trizol Reagent following the manufacturer's instructions. RNA concentration was quantified using a NanoDrop spectrophotometer. Total RNA (2 μg) was reverse‐transcribed using a cDNA Reverse Transcription Kit. Real‐time PCR was performed on a Bio‐Rad system using SYBR Green Master Mix. Primer sequences are listed in Table S2.

### 2.9 RNA sequencing

The RNA quality was examined using a NanoDrop spectrophotometer. RNA sequencing, library construction, and analysis were performed by Sinotech Genomics Co. Ltd. (Shanghai, China). |Log2FC| ≥0.58 and *p*‐value <0.05 were used to determine differential genes.

### 2.10 Statistical analysis

Data are represented as means ± SEM. Groups were compared using the unpaired, two‐tailed Student's *t*‐test. *p* < 0.05 were considered statistically significant.

## 3 RESULTS

### 3.1 FAM20C is upregulated in white adipose tissue (WAT) of obese mice

**FIGURE 1:**
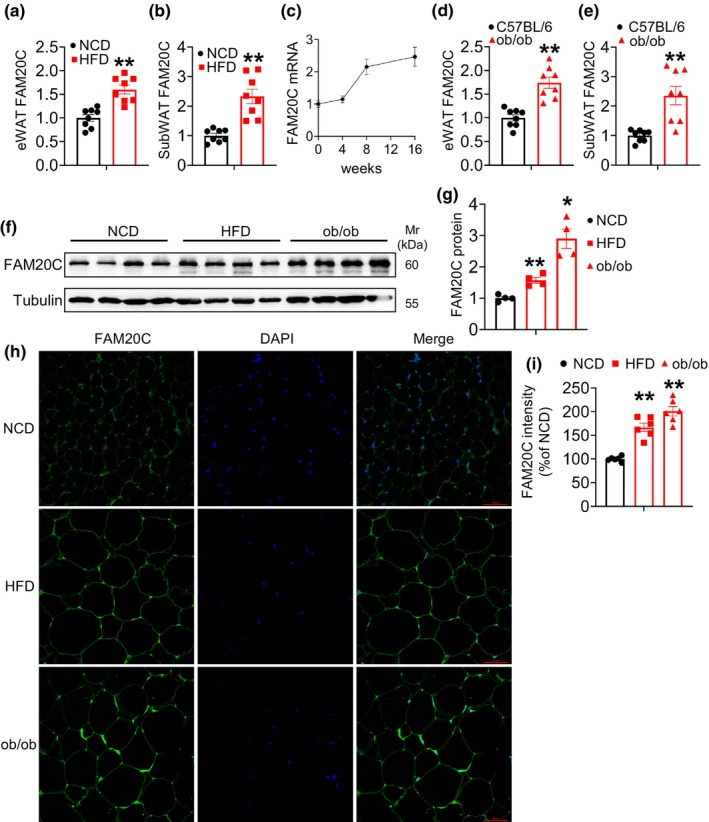
FAM20C was increased in adipose tissue with obesity. (a, b) Gene expression levels in epididymal white adipose tissue (eWAT) and subcutaneous white adipose tissue (subWAT) of mice fed an HFD. (c) Gene expression after HFD fed under indicated time. (d, e) The gene expression levels in the eWAT and subWAT of ob/ob mice compared to control mice. (f, g) Western blot analysis of FAM20C expression in eWAT of 12 weeks fed HFD or ob/ob mice relative to normal chow diet‐fed mice. (h, i) eWAT section from 12 weeks HFD fed mice or ob/ob mice and normal chow diet was stained with anti‐FAM20C antibody. Scale bars: 100 μm. Data are showed as mean ± SEM. *p < 0.05, **p < 0.01.

After 12 weeks of high‐fat diet (HFD) exposure, the transcription levels of FAM20C were significantly increased in the WAT of mice compared to those on a normal chow diet (NCD) (Figure 1a–c). Elevated FAM20C expression was also confirmed in the WAT of obese (ob/ob) mice (Figure 1d,e). The protein level of FAM20C in WAT from obese mice was also increased (Figure 1f,g). Immunofluorescence staining further validated the upregulation of FAM20C in the epididymal WAT (eWAT) of obese mice (Figure 1h,i). These results suggest that FAM20C plays a role in regulating adipocytes during lipid loading, prompting further investigation into its function in adipose tissue in vivo.

### 3.2 Adipocyte‐specific FAM20C knockout mice show reduce adiposity during obesity

**FIGURE 2:**
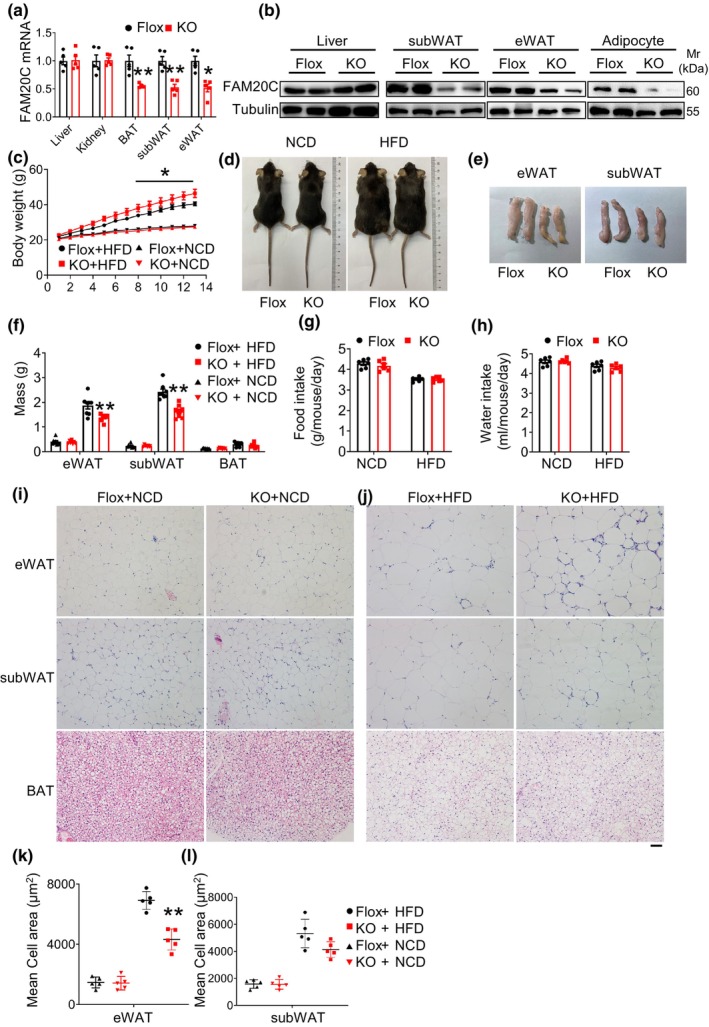
Adipose‐specific FAM20C deletion protects mice to obesity, impaired adipose tissue expansion. (a) qPCR analysis of FAM20C mRNA expression in adipose tissue from mice. (b) Western blot analysis of FAM20C protein level in tissues and mature adipocyte from control and KO mice. (c) Body weight of control and KO mice fed an NCD and HFD. (d) Representative pictures of control and KO mice. (e) Representative WAT pictures of control and KO mice. (f) Weight of adipose tissue including eWAT, subWAT and BAT. (g) Food intake. (h) Water intake. (i) Hematoxylin and eosin staining of eWAT, subWAT and BAT of NCD fed control and KO mice. Scale bar = 100 μm. (j) Hematoxylin and eosin staining of eWAT, subWAT and BAT of HFD‐fed control and KO mice. Scale bar = 100 μm. (k) eWAT adipocyte size of control and KO mice. (l) subWAT adipocyte size of control and KO mice. Data are represented as mean ± SEM. *p < 0.05, **p < 0.01.

To investigate the potential role of FAM20C in the development of obesity, we generated adipocyte‐specific FAM20C deficient mice by crossing FAM20C flox/flox mice with Adipoq‐cre transgenic mice (Figure S1). As anticipated, FAM20C mRNA levels were significantly reduced in WAT, including eWAT, subcutaneous WAT (subWAT), and brown adipose tissue (BAT), but not in other major tissue (Figure 2a). Western blot results verified FAM20C deletion in WAT and mature adipocyte but not in the liver or kidney (Figure 2b). These findings collectively validate the adipocyte‐specific deletion of FAM20C in the KO mice.

To assess the effect of adipocyte FAM20C deficiency on obesity, 6‐week‐old mice were fed an NCD or HFD. Although body weight remained unaffected by NCD, it was notably reduced in the HFD group (Figure 2c,d). We found that the white adipose tissue weight including eWAT and subWAT were decreased in response to HFD feeding (Figure 2e,f). KO mice showed no significant differences in food and water intake compared to controls (Figure 2g,h). A decrease in adipose tissue mass can be attributed to a decrease in adipocyte size or number due to abnormal differentiation. Histological analyses showed no differences in WAT and BAT when fed a normal chow diet, but a significant decrease in adipocyte size in the WAT of KO mice was observed when challenged with HFD (Figure 2i,j). Quantification of adipocyte size confirmed the decreased adipocyte size in FAM20C deficient mice in WAT (Figure 2k,l).

### 3.3 Adipose‐specific FAM20C deletion ameliorates insulin resistance

**FIGURE 3:**
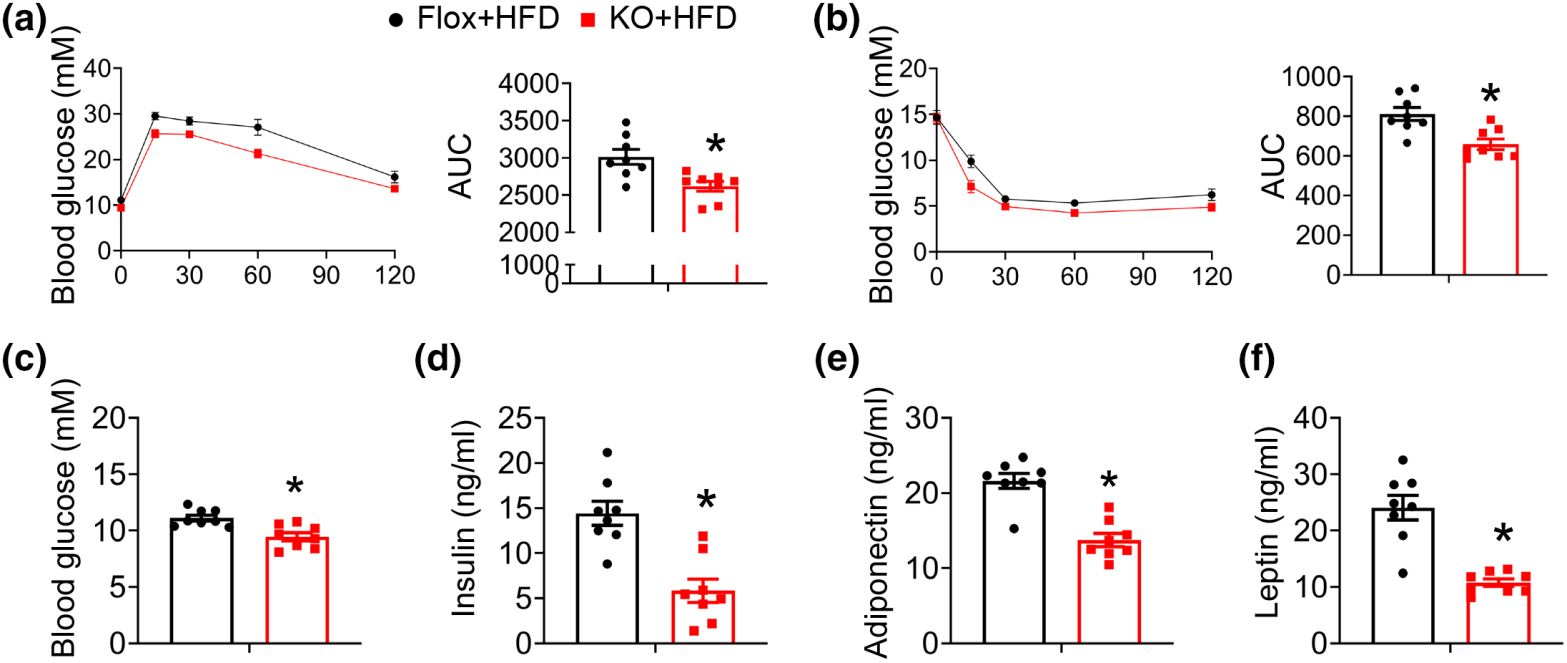
Adipose‐specific FAM20C deletion ameliorates insulin resistance. (a) Blood glucose concentrations during glucose tolerance tests from HFD fed mice. (b) Blood glucose concentrations during insulin tolerance tests from HFD fed mice. (c) Fasting blood glucose and (d) insulin concentration after 12 weeks HFD feeding. (e) Serum adiponectin and (f) leptin concentration after 12 weeks HFD feeding. Data are represented as mean ± SEM. *p < 0.05.

Obesity is associated with impaired glucose homeostasis and insulin resistance. To evaluate the impact of adipocyte‐specific FAM20C deletion on these parameters, we performed glucose tolerance tests (GTT) and insulin tolerance tests (ITT) on mice fed an NCD. There were no statistically significant differences in glucose tolerance or insulin sensitivity on NCD. However, under HFD conditions, KO mice exhibited improved glucose and insulin tolerance (Figure 3a,b). HFD‐fed KO mice showed significantly decreased fasting glucose (Figure 3c) and serum insulin levels (Figure 3d). Serum adiponectin and leptin levels were both decreased in KO mice compared to controls under HFD conditions (Figure 3e,f).

### 3.4 Loss of FAM20C in adipocyte increases energy expenditure

**FIGURE 4:**
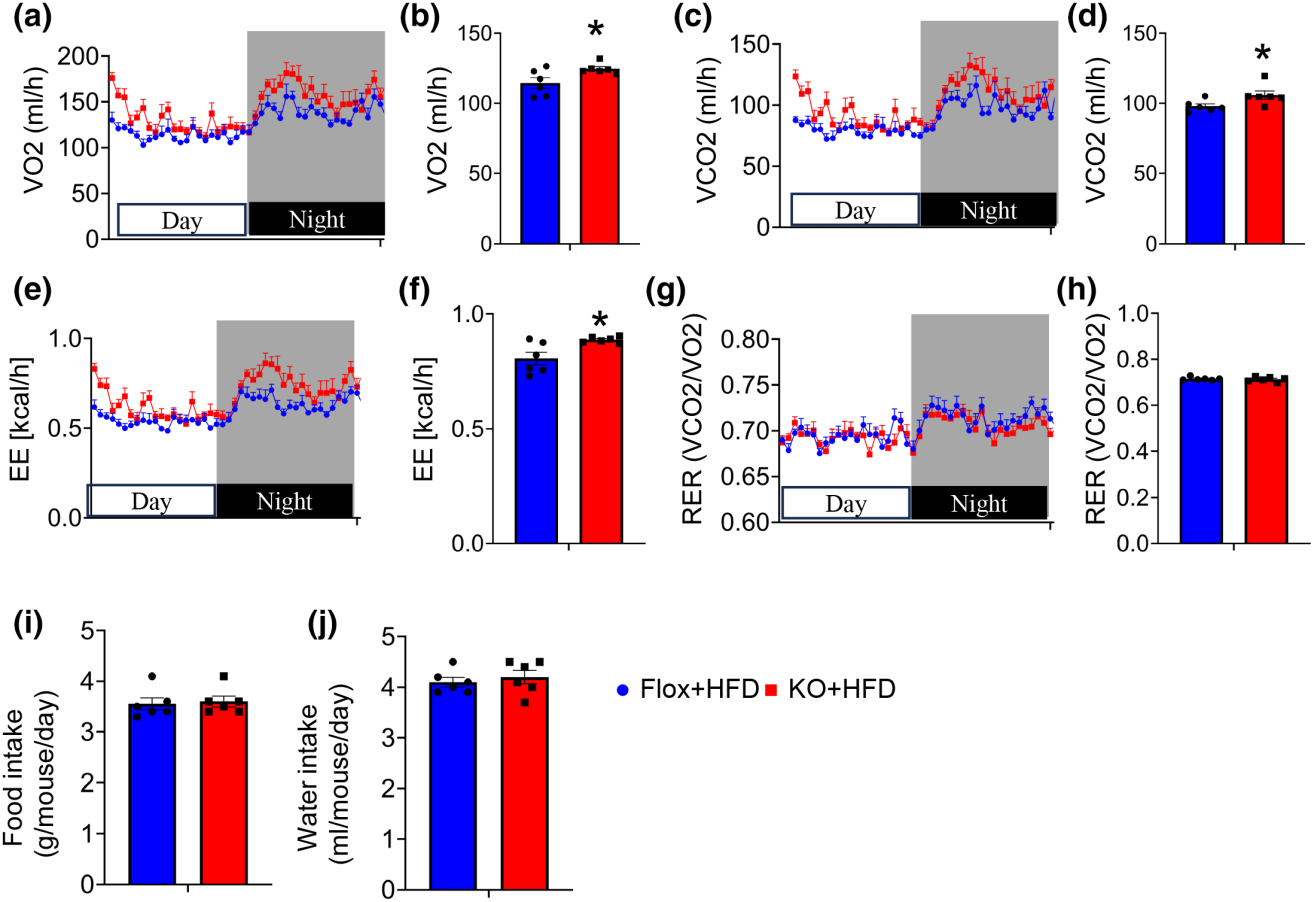
Adipose‐specific FAM20C deletion increase energy expenditure. (a–h) Indirect calorimetry analysis. VO_2_: Rate of oxygen consumption; VCO_2_: Rate of carbon dioxide production. EE: Energy expenditure; RER: Respiratory exchange ratio; *n* = 6/group. (i, j) Monitoring of food intake and water intake. Data are represented as mean ± SEM. *p < 0.05.

We next examined whether adipocyte deletion of FAM20C affects energy balance. Indirect calorimetry experiment showed that VO_2_ and VCO_2_ were both increased in KO mice compared to the control group under HFD condition (Figure 4a–d), indicating increased energy expenditure (Figure 4e,f). Notably, the respiratory exchange ratio (RER) values were similar between the control and KO mice (Figure 4g,h). To exclude the effect of energy intake, we measured the water and food intake. Results showed that daily water consumption and food intake has no significant differences between the control and KO mice (Figure 4i,j).

### 3.5 HFD‐fed FAM20C KO mice show ameliorated hepatic steatosis

**FIGURE 5:**
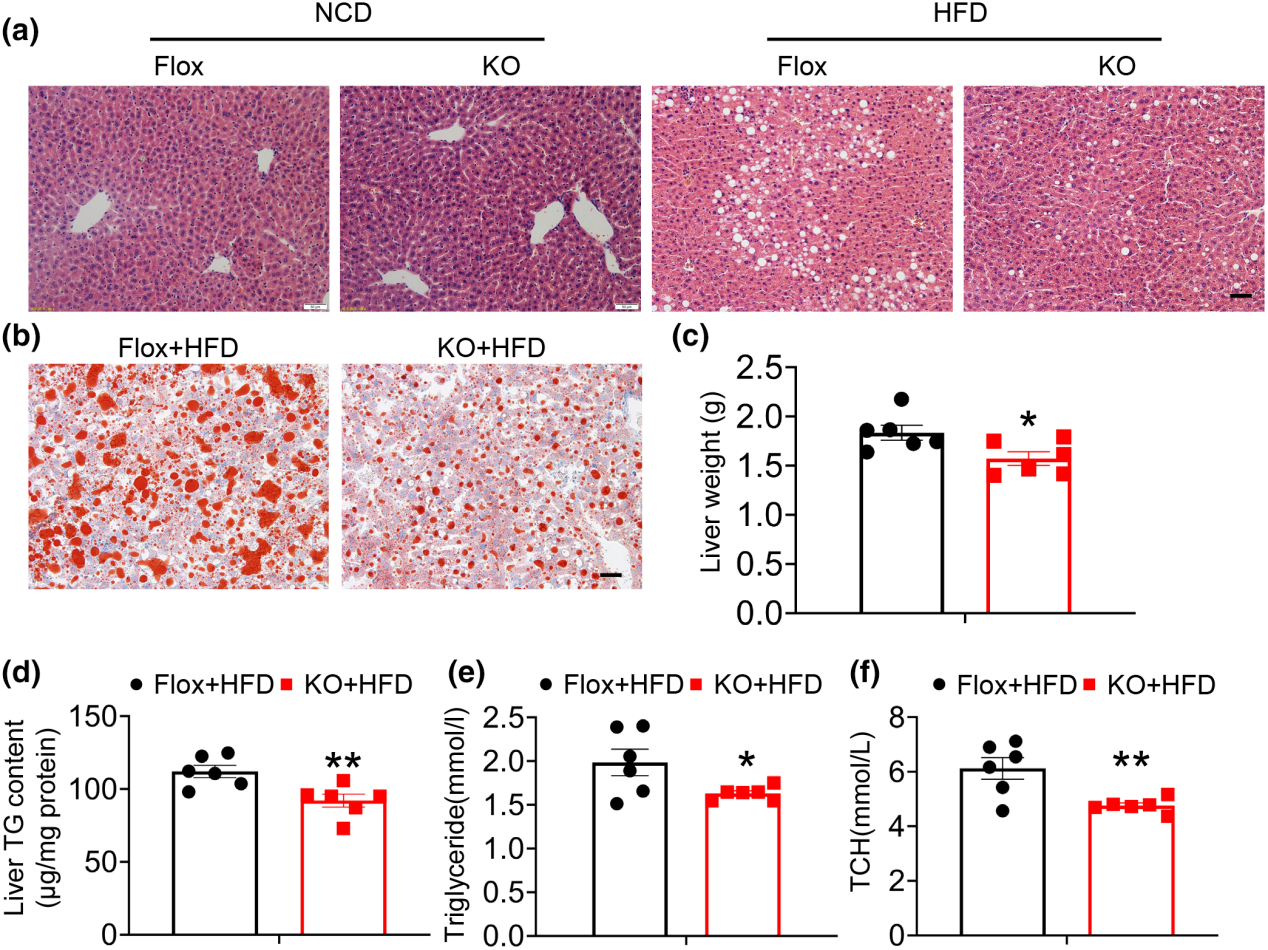
HFD‐fed adipocyte‐specific FAM20C KO mice ameliorates hepatosis. (a) Hematoxylin and eosin sections of liver tissues from NCD and HFD mice. Scale bar: 100 μm. (b) Oil‐red O staining of liver tissues from HFD‐induced mice. Scale bar: 100 μm. (c) liver weight from HFD fed control and KO mice. (d) Hepatic triglycerides level in livers of control and KO mice fed an HFD. (e) Level of serum Triglyceride and (f) total cholesterol from control and KO HFD‐fed mice. Data are represented as mean ± SEM. *p < 0.05, **p < 0.01.

Given the correlation between hepatic steatosis, obesity, and insulin resistance, we examined the effect of adipose FAM20C deletion on liver lipid accumulation under HFD. Mice fed an HFD exhibited greater liver lipid accumulation compared to those on an NCD, whereas KO mice exhibited reduced liver lipid accumulation under HFD (Figure 5a,b). The liver weight of HFD‐fed KO mice was also lower than controls (Figure 5c). Hepatic triglyceride levels were markedly lower in KO mice (Figure 5d). Serum triglyceride and total cholesterol levels were significantly decreased in KO mice (Figure 5e,f). These findings suggest that adipose‐specific FAM20C deficiency ameliorates hepatic steatosis.

### 3.6 Loss of FAM20C in adipose tissue decreases inflammation and fibrosis in adipose tissue

**FIGURE 6:**
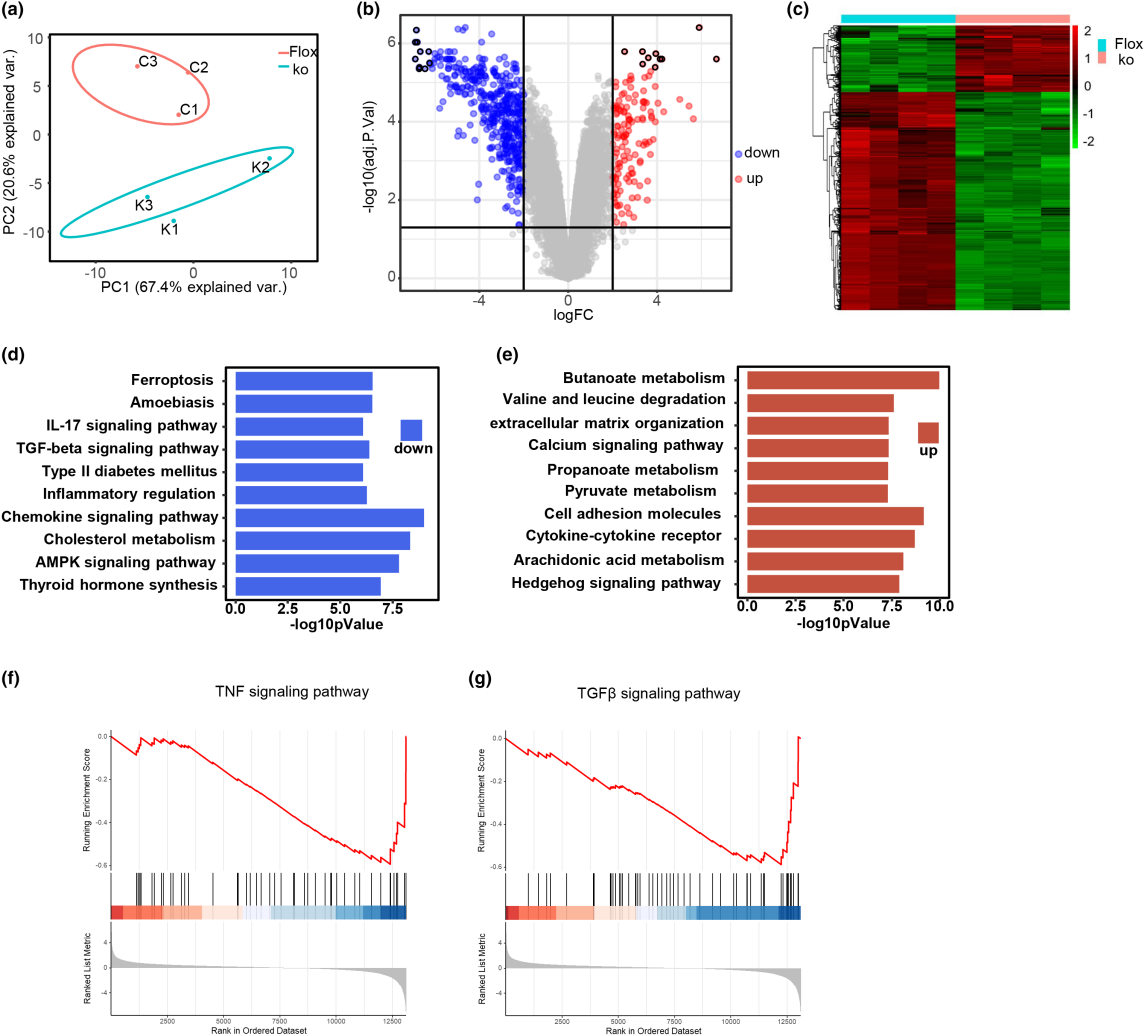
Gene profile of WAT from HFD‐fed adipocyte‐specific FAM20C KO mice and control mice. (a) The principal components analysis (PCA) (b, c) Heatmap and volcano plot analysis of RNA sequence in WAT of FAM20C KO and control mice; *n* = 4/group. (d, e) KEGG pathway analysis of differentially expressed genes (DEGs) of FAM20C KO mice and control mice. (f, g) Gene Set Enrichment Analysis (GSEA) identifying the significantly downregulated pathways.

**FIGURE 7:**
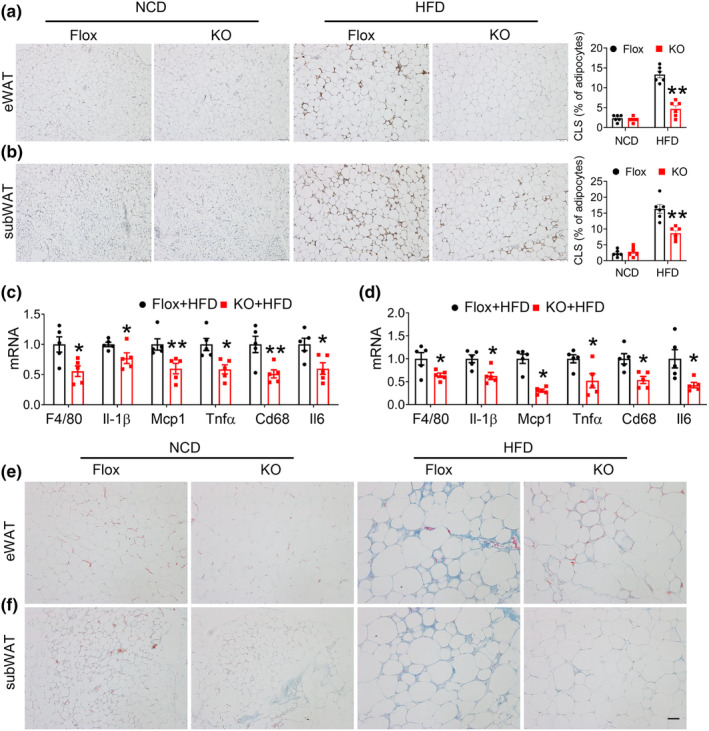
Deletion of FAM20C in adipocyte ameliorates collagen deposition and macrophage infiltration in adipose tissue. (a, b) Immunohistochemistry for F4/80 in eWAT and subWAT from NCD or HFD‐fed control and KO mice and quantification, Scale bar = 100 μm. (c, d) qPCR analysis of inflammatory genes in adipose tissue from eWAT and subWAT of HFD‐fed mice. (e, f) Trichrome staining for collagen deposition in eWAT and subWAT from NCD or HFD‐fed control and KO mice. Data are represented as mean ± SEM. *p < 0.05, **p < 0.01.

To explore the potential mechanisms by which FAM20C deletion affects adipose tissue, we performed RNA sequencing of WAT from control and KO mice fed an HFD for 12 weeks (Figure 6a–c). KEGG pathway analysis revealed that genes associated with inflammation and fibrosis pathways were significantly decreased in KO mice (Figure 6d,e). Gene set enrichment analysis (GSEA) showed suppressed TNF and TGFβ signaling pathways in the WAT of KO mice (Figure 6f,g). Under HFD conditions, adipose tissue inflammation, marked by macrophage recruitment, was significantly ameliorated in KO mice, evidenced by decreased crown‐like structures (Figure 7a,b). This was supported by reduced mRNA levels of macrophage markers and key proinflammatory cytokines, including TNF‐α and MCP‐1 (Figure 7c,d). Fibrosis, observed to a lesser degree with Masson's trichrome staining, was also reduced in WAT from HFD‐fed KO mice (Figure 7e,f). These findings demonstrate that loss of FAM20C in adipose tissues ameliorates metabolic dysfunction, inflammation, and fibrosis.

## 4 DISCUSSION

Adipose tissue is crucial for maintaining metabolic processes and energy balance. In this study, we observed elevated FAM20C levels in adipocytes under obese conditions. Adipocyte‐specific FAM20C deletion conferred resistance to HFD‐induced obesity in mice. Through analyses of tissues from control and mutant animals, we established a critical new role for FAM20C proteins in the regulation of adipose tissue homeostasis. Our findings revealed a novel role for FAM20C, diverging from its previously recognized functions in bone development and cancer progression.

To date, studies on FAM20C have primarily focused on its role in bone development and malignancies. For instance, overexpression of FAM20C in osteoblasts increases bone mass, and its inactivation in the dental epithelium results in dentin and alveolar bone defects (Wang et al., 2015). FAM20C is upregulated in triple‐negative breast cancer and may play a critical role in tumor invasion and metastasis, with Fam20C knockout cancer cells showing less malignant invasion (Tagliabracci et al., 2015). Our study unveiled a novel function of adipocyte FAM20C signaling in regulating adipose tissue homeostasis and glucose metabolism. The deletion of FAM20C had a minor impact on glucose metabolism in mice fed a normal chow diet (NCD). However, in mice fed an HFD, deletion of FAM20C in adipocytes led to significant improvements in metabolic abnormalities, including decreased fasting blood glucose levels, enhanced glucose tolerance, improved insulin sensitivity, and reduced fatty liver. These results suggest that altered FAM20C expression in adipocytes may contribute to the development of metabolic diseases such as type 2 diabetes, obesity, and metabolic syndrome in humans. Therefore, our study highlights the importance of investigating adipocyte FAM20C signaling in human diseases.

FAM20C proteins, which respond to extracellular matrix (ECM) stimulation, have been implicated in integrin signaling (Wang et al., 2010). Deficiency of ECM components, such as matrix metalloproteinase 14 (MMP14), collagen Va3, or tissue inhibitor of matrix metalloproteinase 2, impairs glucose homeostasis in vivo (Chun et al., 2006; Henegar et al., 2008; Huang et al., 2011; Jaworski et al., 2011). Results from in vitro study have shown that ECM stiffness regulates adipogenesis (Liu et al., 2005). While the expression of FAM20C in adipose tissue did not regulate adiposity and glucose metabolism under NCD, the differences observed were likely too subtle to alter overall body metabolism. However, significant improvements in glucose homeostasis were observed under HFD conditions. It is plausible that, under conditions of lower metabolic stress, adipocytes may better cope with imbalances.

In conclusion, we demonstrated that adipocyte FAM20C plays a pivotal role in the regulation of adipocyte expansion and glucose metabolism. Deletion of FAM20C mitigates adipocyte enlargement and improves metabolic dysfunction during HFD challenge. This study identified a novel role of FAM20C in linking adipocyte signaling and glucose homeostasis. Our findings suggest that FAM20C is a potential target for treating obesity and associated metabolic disorders.

## 5 LIMITATIONS OF STUDY

Our study has some limitations. Given the critical role of FAM20C in adipose tissue metabolism under nutritional stress, further investigation is needed to examine its effects on adipocyte differentiation in vitro. Additionally, we need to explore the impact of FAM20C on mature adipocyte metabolism using a Cre‐ERT mouse model. Moreover, to rule out the effects of estrogen, we used male mice in the current study. It also needs to be proven that the effect of FAM20c on adiposity in female mice.

## AUTHOR CONTRIBUTIONS

Study design: X. H. and L. D. Study conduct and data collection and analysis: L. D., Y. H., F. Z., and P. C. Data interpretation: X. H., L. D., Y. H., F. Z., and P. C. Drafting of the manuscript: X. H. and L. D. X. H. and L. D. took responsibility for the integrity of the data analysis.

## CONFLICT OF INTEREST STATEMENT

The authors declare that they have no conflicts of interest.

## Supporting information


Figure S1.

Data S1.

## Data Availability

Data can be made available upon reasonable request to the corresponding author.

## References

[phy270126-bib-0001] Appleton, S. L. , Seaborn, C. J. , Visvanathan, R. , Hill, C. L. , Gill, T. K. , Taylor, A. W. , Adams, R. J. , & North West Adelaide Health Study Team . (2013). Diabetes and cardiovascular disease outcomes in the metabolically healthy obese phenotype: A cohort study. Diabetes Care, 36(8), 2388–2394. 23491523 10.2337/dc12-1971 PMC3714523

[phy270126-bib-0002] Chun, T. H. , Hotary, K. B. , Sabeh, F. , Saltiel, A. R. , Allen, E. D. , & Weiss, S. J. (2006). A pericellular collagenase directs the 3‐dimensional development of white adipose tissue. Cell, 125(3), 577–591. 16678100 10.1016/j.cell.2006.02.050

[phy270126-bib-0003] Denis, G. V. , & Obin, M. S. (2013). 'Metabolically healthy obesity: Origins and implications. Molecular Aspects of Medicine, 34(1), 59–70. 23068072 10.1016/j.mam.2012.10.004 PMC3583231

[phy270126-bib-0004] Eames, B. F. , Yan, Y. L. , Swartz, M. E. , Levic, D. S. , Knapik, E. W. , Postlethwait, J. H. , & Kimmel, C. B. (2011). Mutations in fam20b and xylt1 reveal that cartilage matrix controls timing of endochondral ossification by inhibiting chondrocyte maturation. PLoS Genetics, 7(8), e1002246. 21901110 10.1371/journal.pgen.1002246 PMC3161922

[phy270126-bib-0005] Gonzalez‐Muniesa, P. , Martinez‐Gonzalez, M. A. , Hu, F. B. , Despres, J. P. , Matsuzawa, Y. , Loos, R. J. F. , et al. (2017). Obesity. Nature Reviews. Disease Primers, 3, 17034. 10.1038/nrdp.2017.34 28617414

[phy270126-bib-0006] Hammarstedt, A. , Gogg, S. , Hedjazifar, S. , Nerstedt, A. , & Smith, U. (2018). Impaired adipogenesis and dysfunctional adipose tissue in human hypertrophic obesity. Physiological Reviews, 98(4), 1911–1941. 30067159 10.1152/physrev.00034.2017

[phy270126-bib-0007] Henegar, C. , Tordjman, J. , Achard, V. , Lacasa, D. , Cremer, I. , Guerre‐Millo, M. , Poitou, C. , Basdevant, A. , Stich, V. , Viguerie, N. , Langin, D. , Bedossa, P. , Zucker, J. D. , & Clement, K. (2008). Adipose tissue transcriptomic signature highlights the pathological relevance of extracellular matrix in human obesity. Genome Biology, 9(1), R14. 18208606 10.1186/gb-2008-9-1-r14 PMC2395253

[phy270126-bib-0009] Huang, G. , Ge, G. , Wang, D. , Gopalakrishnan, B. , Butz, D. H. , Colman, R. J. , Nagy, A. , & Greenspan, D. S. (2011). alpha3(V) collagen is critical for glucose homeostasis in mice due to effects in pancreatic islets and peripheral tissues. The Journal of Clinical Investigation, 121(2), 769–783. 21293061 10.1172/JCI45096 PMC3026738

[phy270126-bib-0010] Jaworski, D. M. , Sideleva, O. , Stradecki, H. M. , Langlois, G. D. , Habibovic, A. , Satish, B. , Tharp, W. G. , Lausier, J. , LaRock, K. , Jetton, T. L. , Peshavaria, M. , & Pratley, R. E. (2011). Sexually dimorphic diet‐induced insulin resistance in obese tissue inhibitor of metalloproteinase‐2 (TIMP‐2)‐deficient mice. Endocrinology, 152(4), 1300–1313. 21285317 10.1210/en.2010-1029 PMC3060627

[phy270126-bib-0011] Krotkiewski, M. , Bjorntorp, P. , Sjostrom, L. , & Smith, U. (1983). Impact of obesity on metabolism in men and women. Importance of regional adipose tissue distribution. The Journal of Clinical Investigation, 72(3), 1150–1162. 6350364 10.1172/JCI111040 PMC1129283

[phy270126-bib-0012] Lee, K. Y. , Russell, S. J. , Ussar, S. , Boucher, J. , Vernochet, C. , Mori, M. A. , Smyth, G. , Rourk, M. , Cederquist, C. , Rosen, E. D. , Kahn, B. B. , & Kahn, C. R. (2013). Lessons on conditional gene targeting in mouse adipose tissue. Diabetes, 62(3), 864–874. 23321074 10.2337/db12-1089 PMC3581196

[phy270126-bib-0013] Liu, C. , Zhang, H. , Jani, P. , Wang, X. , Lu, Y. , Li, N. , Xiao, J. , & Qin, C. (2018). FAM20C regulates osteoblast behaviors and intracellular signaling pathways in a cell‐autonomous manner. Journal of Cellular Physiology, 233(4), 3476–3486. 28926103 10.1002/jcp.26200 PMC5741497

[phy270126-bib-0014] Liu, J. , DeYoung, S. M. , Zhang, M. , Zhang, M. , Cheng, A. , & Saltiel, A. R. (2005). Changes in integrin expression during adipocyte differentiation. Cell Metabolism, 2(3), 165–177. 16154099 10.1016/j.cmet.2005.08.006

[phy270126-bib-0015] Liu, P. , Ma, S. , Zhang, H. , Liu, C. , Lu, Y. , Chen, L. , & Qin, C. (2017). Specific ablation of mouse Fam20C in cells expressing type I collagen leads to skeletal defects and hypophosphatemia. Scientific Reports, 7(1), 3590. 28620244 10.1038/s41598-017-03960-x PMC5472603

[phy270126-bib-0017] Monji, A. , Zhang, Y. , Kumar, G. V. N. , Guillermier, C. , Kim, S. , Olenchock, B. , & Steinhauser, M. L. (2022). A cycle of inflammatory adipocyte death and regeneration in murine adipose tissue. Diabetes, 71(3), 412–423. 35040481 10.2337/db20-1306 PMC8893943

[phy270126-bib-0018] Nalbant, D. , Youn, H. , Nalbant, S. I. , Sharma, S. , Cobos, E. , Beale, E. G. , Du, Y. , & Williams, S. C. (2005). FAM20: An evolutionarily conserved family of secreted proteins expressed in hematopoietic cells. BMC Genomics, 6, 11. 15676076 10.1186/1471-2164-6-11 PMC548683

[phy270126-bib-0019] Neeland, I. J. , Ross, R. , Despres, J. P. , Matsuzawa, Y. , Yamashita, S. , Shai, I. , Seidell, J. , Magni, P. , Santos, R. D. , Arsenault, B. , Cuevas, A. , Hu, F. B. , Griffin, B. , Zambon, A. , Barter, P. , Fruchart, J. C. , & Eckel, R. H. (2019). Visceral and ectopic fat, atherosclerosis, and cardiometabolic disease: A position statement. The Lancet Diabetes and Endocrinology, 7(9), 715–725. 31301983 10.1016/S2213-8587(19)30084-1

[phy270126-bib-0020] Ryden, M. , Andersson, D. P. , Bergstrom, I. B. , & Arner, P. (2014). Adipose tissue and metabolic alterations: Regional differences in fat cell size and number matter, but differently: A cross‐sectional study. The Journal of Clinical Endocrinology and Metabolism, 99(10), E1870–E1876. 24937536 10.1210/jc.2014-1526

[phy270126-bib-0021] Spalding, K. L. , Arner, E. , Westermark, P. O. , Bernard, S. , Buchholz, B. A. , Bergmann, O. , Blomqvist, L. , Hoffstedt, J. , Näslund, E. , Britton, T. , Concha, H. , Hassan, M. , Rydén, M. , Frisén, J. , & Arner, P. (2008). Dynamics of fat cell turnover in humans. Nature, 453(7196), 783–787. 18454136 10.1038/nature06902

[phy270126-bib-0022] Tagliabracci, V. S. , Wiley, S. E. , Guo, X. , Kinch, L. N. , Durrant, E. , Wen, J. , Xiao, J. , Cui, J. , Nguyen, K. B. , Engel, J. L. , Coon, J. J. , Grishin, N. , Pinna, L. A. , Pagliarini, D. J. , & Dixon, J. E. (2015). A single kinase generates the majority of the secreted Phosphoproteome. Cell, 161(7), 1619–1632. 26091039 10.1016/j.cell.2015.05.028 PMC4963185

[phy270126-bib-0023] Voulgari, C. , Tentolouris, N. , Dilaveris, P. , Tousoulis, D. , Katsilambros, N. , & Stefanadis, C. (2011). Increased heart failure risk in normal‐weight people with metabolic syndrome compared with metabolically healthy obese individuals. Journal of the American College of Cardiology, 58(13), 1343–1350. 21920263 10.1016/j.jacc.2011.04.047

[phy270126-bib-0024] Wang, X. , Hao, J. , Xie, Y. , Sun, Y. , Hernandez, B. , Yamoah, A. K. , Prasad, M. , Zhu, Q. , Feng, J. Q. , & Qin, C. (2010). Expression of FAM20C in the osteogenesis and odontogenesis of mouse. The Journal of Histochemistry and Cytochemistry, 58(11), 957–967. 20644212 10.1369/jhc.2010.956565 PMC2958138

[phy270126-bib-0025] Wang, X. , Wang, J. , Liu, Y. , Yuan, B. , Ruest, L. B. , Feng, J. Q. , & Qin, C. (2015). The specific role of FAM20C in dentinogenesis. Journal of Dental Research, 94(2), 330–336. 25515778 10.1177/0022034514563334 PMC4300304

[phy270126-bib-0026] Wang, X. , Wang, S. , Li, C. , Gao, T. , Liu, Y. , Rangiani, A. , Sun, Y. , Hao, J. , George, A. , Lu, Y. , Groppe, J. , Yuan, B. , Feng, J. Q. , & Qin, C. (2012). Inactivation of a novel FGF23 regulator, FAM20C, leads to hypophosphatemic rickets in mice. PLoS Genetics, 8(5), e1002708. 22615579 10.1371/journal.pgen.1002708 PMC3355082

[phy270126-bib-0027] Xiao, G. , Zhang, T. , Yu, S. , Lee, S. , Calabuig‐Navarro, V. , Yamauchi, J. , Ringquist, S. , & Dong, H. H. (2013). ATF4 protein deficiency protects against high fructose‐induced hypertriglyceridemia in mice. The Journal of Biological Chemistry, 288(35), 25350–25361. 23888053 10.1074/jbc.M113.470526 PMC3757199

[phy270126-bib-0028] Xu, R. , Tan, H. , Zhang, J. , Yuan, Z. , Xie, Q. , & Zhang, L. (2021). Fam20C in human diseases: Emerging biological functions and therapeutic implications. Frontiers in Molecular Biosciences, 8, 790172. 34988120 10.3389/fmolb.2021.790172 PMC8721277

[phy270126-bib-0029] Zuo, H. , Yang, D. , & Wan, Y. (2021). Fam20C regulates bone resorption and breast cancer bone metastasis through Osteopontin and BMP4. Cancer Research, 81(20), 5242–5254. 34433585 10.1158/0008-5472.CAN-20-3328

