# Supplementary figures and images for: Lack of adipocyte FAM20C improves whole body glucose homeostasis
Source: Physiol Rep. 2024 Nov 12;12(21):e70126. doi: 10.14814/phy2.70126 (PMC11557440; doi:10.14814/phy2.70126)

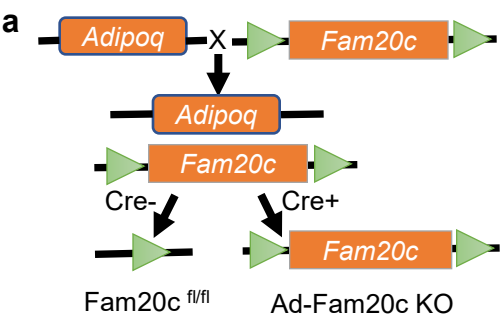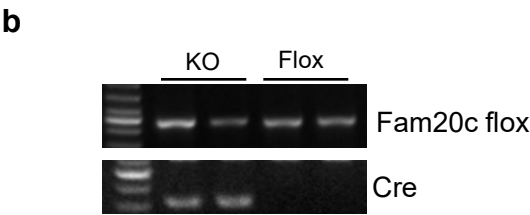

Supplement: Supplementary file 1 — Figure S1. [file PHY2-12-e70126-s001.pdf]
