# Supplementary material for: Lack of adipocyte FAM20C improves whole body glucose homeostasis
Source: Physiol Rep. 2024 Nov 12;12(21):e70126. doi: 10.14814/phy2.70126 (PMC11557440; doi:10.14814/phy2.70126)
Supplement: Supplementary file 2 — Data S1. [file PHY2-12-e70126-s002.docx]

**Supplementary Table 1. Antibody information**

| **Name** | **Supplier** | **Cat no.** | **Application/Dilution** |
| --- | --- | --- | --- |
| Fam20c | Proteintech | 25395-1-AP | WB (1:1000); IF (1:200) |
| F4/80 | Proteintech | 28463-1-AP | IHC (1:200) |
| Tubulin | Cell Signaling Technology Inc | 2128 | WB (1:5000) |

**Supplementary Table 2: Real-time Polymerase Chain Reaction (PCR) Primers**

| Gene Name | Forward | Reverse |
| --- | --- | --- |
| TNFα | CCACGTCGTAGCAAACCACC | GATAGCAAATCGGCTGACGG |
| MCP1 | CACTCACCTGCTGCTACTCA | GCTTGGTGACAAAAACTACAGC |
| IL-1β | TGCCACCTTTTGACAGTGATG | AAGGTCCACGGGAAAGACAC |
| IL-6 | CTCATTCTGCTCTGGAGCCC | CAACTGGATGGAAGTCTCTTGC |
| CD68 | ACTTCGGGCCATGTTTCTCTT | GGGGCTGGTAGGTTGATTGT |
| GAPDH | TTTCTTCTTGCCTTGGGAGA | AGTTCCGCACTTCATTCAGG |
| F4/80 | TGACTCACCTTGTGGTCCTAA | CTTCCCAGAATCCAGTCTTTCC |
| Fam20c | GATGTGACGCGGGATAAGAAG | GCTCGGTGGAACAGTAGTAGG |
